# A high‐fat diet with vitamin D and propylthiouracil produces a pro‐atherogenic phenotype in rats

**DOI:** 10.1002/ame2.70178

**Published:** 2026-04-01

**Authors:** Angélique Lewies, Vitaris Kodogo, Johannes Frederik Wentzel, Francis Edwin Smit

**Affiliations:** ^1^ Department of Cardiothoracic Surgery, Robert WM Frater Cardiovascular Research Centre, Faculty of Health Sciences University of the Free State Bloemfontein South Africa; ^2^ Department of Genetics, Faculty of Natural and Agricultural Sciences University of the Free State Bloemfontein South Africa

**Keywords:** atherosclerosis, dyslipidemia, high‐fat diet (HFD), propylthiouracil (PTU), rat model

## Abstract

**Background:**

Atherosclerosis begins with dyslipidemia, vascular inflammation, and endothelial dysfunction. Rodent models that capture these early events are needed for mechanistic and interventional studies. This study evaluated whether a cholesterol‐rich, high‐fat diet (HFD) supplemented with vitamin D and propylthiouracil (PTU) promotes a pro‐atherogenic phenotype in rats, as evidenced by dyslipidemia, inflammation, markers of endothelial dysfunction, and early vascular remodeling.

**Methods:**

Male Sprague–Dawley rats (*n* = 18) received standard chow or a HFD containing 2% cholesterol, 3% lard, 0.5% cholate, vitamin D (200 000 IU/kg), and PTU (0.2% w/w) for 11 weeks. Terminal serum total cholesterol, high‐density lipoprotein, low‐density lipoprotein (LDL)/very low‐density lipoprotein (VLDL), triglycerides, calcium, interleukin‐6 (IL‐6), C‐reactive protein, serum amyloid A (SAA), circulating endothelial nitric oxide synthase (eNOS), and intracellular adhesion molecule‐1 (ICAM‐1) were measured. The aorta, the coronary arteries, and the liver were examined histologically.

**Results:**

HFD‐fed rats developed significant hypercholesterolemia with higher total cholesterol and LDL/VLDL (*p* < 0.0001) and lower triglycerides (*p* < 0.0001) versus controls. Serum calcium was higher (*p* < 0.0001) without vascular calcification. Aortae exhibited wall thickening, smooth‐muscle disarray, mononuclear infiltrates, and focal foam cell‐like changes; coronary arteries exhibited endothelial irregularities and perivascular infiltrates. Livers exhibited micro‐ and macrovesicular steatosis. IL‐6 and SAA were higher (*p* < 0.05), and circulating eNOS was lower (*p* < 0.05); ICAM‐1 did not differ significantly.

**Conclusion:**

An 11‐week vitamin D/PTU‐supplemented HFD induces an LDL‐dominant dyslipidemia with systemic inflammation and evidence consistent with endothelial dysfunction, alongside histological features of early vascular remodeling and hepatic steatosis. This nongenetic model may be useful for studying early atherogenic changes.

## Graphical abstract

Male Sprague–Dawley rats received a cholesterol‐rich high‐fat diet supplemented with vitamin D and propylthiouracil for 11 weeks. The model induced dyslipidemia (increased total cholesterol/low‐density lipoprotein, decreased triglycerides), inflammation (increased interleukin‐6 and serum amyloid A), hypercalcemia, and reduced circulating endothelial nitric oxide synthase. Histology revealed aortic wall thickening with smooth‐muscle disorganization, coronary endothelial irregularities with perivascular infiltrates, and hepatic steatosis, consistent with a pro‐atherogenic phenotype in a nongenetic rat model. Created using BioRender.com.

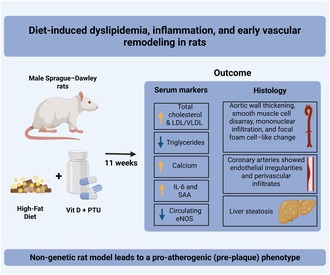

## 1 INTRODUCTION

Atherosclerosis results from the interplay of dyslipidemia, inflammation, and endothelial dysfunction.^1^, ^2^, ^3^ Hypercholesterolemia promotes oxidative stress and pro‐inflammatory cytokine release, whereas endothelial dysfunction, marked by reduced nitric oxide (NO) bioavailability and increased adhesion molecules (ICAM‐1, Vascular cell adhesion molecule, VCAM‐1), is a critical early step in atherogenesis.^2^, ^3^ Elevated serum amyloid A (SAA) and interleukin‐6 (IL‐6) further amplify vascular inflammation.^4^, ^5^, ^6^, ^7^ Particularly, SAA appears to be causal as well as a biomarker, inducing endothelial adhesion molecules (E‐selectin, ICAM‐1, VCAM‐1) via Toll‐like receptor 2 (TLR2) in vitro.^5^

Nongenetic rodent models are essential for studying these mechanisms.^8^ In rats, a cholesterol‐rich high‐fat diet (HFD) combined with vitamin D induces plaque in the aortic root but typically not in the coronary arteries, likely reflecting the high rodent heart rate.^8^, ^9^ Propylthiouracil (PTU), a thyroid inhibitor that lowers heart rate, has been shown to promote coronary atherosclerosis in rats.^10^, ^11^, ^12^ Therefore, HFD models incorporating vitamin D, with or without PTU, with vitamin D administered intraperitoneally (i.p.), are widely used in intervention studies.^13^, ^14^, ^15^

Classic hypervitaminosis‐D protocols administer vitamin D_3_ intramuscularly at 300 000 IU/kg once or subcutaneously at 0, 24, and 48 h,^16^, ^17^ or at 600 000 IU/kg i.p. once,^18^ with two repeated doses of 300 000 IU/kg i.p.^19^ Dietary models have historically used very high doses of vitamin D_2_ (>1 000 000 IU/kg diet), with cholesterol/cholate to accelerate atherosclerotic arteriosclerosis.^20^ However, excessive vitamin D supplementation also led to increased mortality.^16^, ^20^ We therefore employed a moderate‐dose combination to mimic pre‐plaque endothelial dysfunction and vascular remodeling with improved tolerability. This study evaluated whether an HFD containing cholesterol, lard, cholate, vitamin D (200 000 IU/kg diet), and PTU (0.2%) induces a pro‐atherogenic phenotype characterized by dyslipidemia, inflammation, signs of endothelial dysfunction, and histopathological changes in the aorta and coronary arteries.

## 2 MATERIALS AND METHODS

### 2.1 Materials

Male Sprague–Dawley rats were bred by the Vivarium of the Preclinical Drug Development Platform at the North‐West University, Potchefstroom, South Africa, and subsequently sourced by the Animal Research Centre (ARC), University of the Free State (UFS), South Africa. The diets were purchased from Specialty Feeds (Glen Forrest, Western Australia). The HFD was prepared by supplementing the basal meat‐free rat and mouse diet with 2% cholesterol, 3% lard, 0.5% cholate (bile salt), 0.2% PTU, and 200 000 IU vitamin D_3_ per kilogram of diet. Vitamin D_3_ and PTU were incorporated directly into the pelleted diet. The control group received an unsupplemented basal diet. Detailed diet composition is presented in the supplementary information.

### 2.2 Animal housing and routine care

All animal‐related experimental procedures were conducted in accordance with the Animals in Research: Reporting In Vivo Experiments (ARRIVE) guidelines^21^ and the South African National Standard for the care and use of animals for scientific purposes (SANS 10386:2021). Eight‐week‐old male Sprague–Dawley rats (*n* = 18) were used in this study. Rats were housed in a temperature‐ and humidity‐controlled environment (22 ± 2°C, 55% ± 15% relative humidity) with a 12‐h light–dark cycle at the ARC, UFS, Bloemfontein. Two rats were allocated per polysulfone cage (56 × 35 × 20 cm, width × depth × height) with pine wood shavings as bedding.

### 2.3 Randomization, allocation concealment, and blinding

Eighteen 8‐week‐old male Sprague–Dawley rats were randomly allocated to either a control diet (*n* = 8) or a HFD (*n* = 10) group by an animal laboratory technician not involved in outcome assessments. Allocation was performed at the individual‐animal level, after which animals were housed two per cage, resulting in four control and five HFD cages (two rats per cage). Diet administration and routine monitoring were conducted unblinded due to the nature of the intervention. To reduce assessment bias, samples and outcome data were labeled using randomization codes, and histology scoring and downstream data analysis were performed blinded to group assignment until completion of analyses.

### 2.4 Feeding experiments

**FIGURE 1:**
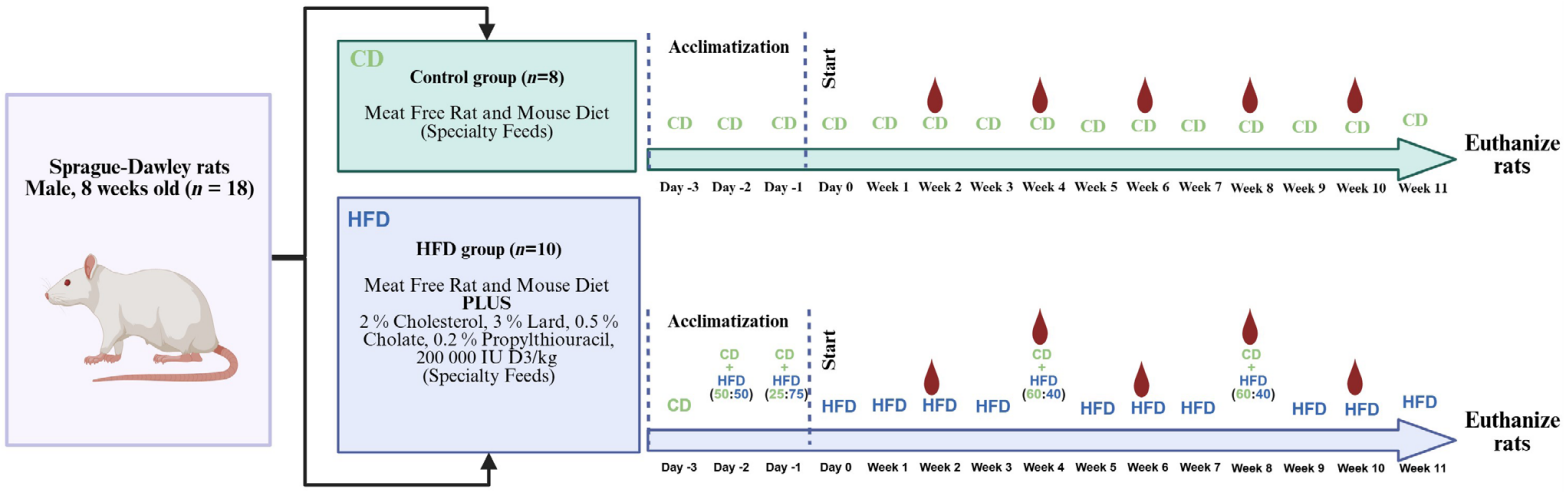
Outline for feeding experiments. The HFD group received a full atherogenic diet for 9 weeks over an 11‐week protocol, with weeks 4 and 8 given as a 60:40 (control–HFD) mix to prevent severe weight loss. CD, control diet; HFD, high‐fat diet (created using BioRender.com).

The feeding experiment design is shown in Figure 1; rats had ad libitum access to demineralized water. Each cage received 50 g of the respective feed daily (25 g per rat); uneaten food was weighed and replaced with fresh feed each day. Rats were transitioned to the HFD over 3 days (day 1: 50% chow/50% HFD, day 2: 25% chow/75% HFD, day 3: 100% HFD). This gradual transition minimized feed refusal and supported dietary adaptation. After acclimatization, rats in the HFD group were maintained on the HFD for 11 weeks; to prevent severe weight loss, the regimen included intermittent dilution weeks, in which animals alternated 3 weeks of full HFD with 1 week of a 60:40 control–HFD mix. This resulted in 9 weeks of full‐strength HFD exposure with two welfare‐driven, reduced‐strength weeks. Body weight was monitored weekly. Whole‐blood lipid monitoring was performed biweekly up to week 10, with terminal serum lipid analysis performed when the animals were euthanized. Blood samples were collected biweekly from the lateral saphenous vein for cholesterol and triglyceride measurements using the Mission 3‐in‐1 Cholesterol Meter (ACON Laboratories, Inc., San Diego, CA) with Mission Cholesterol Test 3–1 Lipid Panel Strips (cholesterol range: 100–500 mg/dL, triglycerides: 45–650 mg/dL). At the end of the 11‐week period, terminal blood samples were collected under 4% isoflurane anesthesia, and rats were euthanized using sodium pentobarbital while maintained under isoflurane sedation. Serum was separated, aliquoted, and stored at −80°C for subsequent analyses.

### 2.5 Serum cholesterol and triglyceride measurements

Terminal serum total cholesterol, high‐density lipoprotein (HDL) cholesterol, and the combined low‐density lipoprotein (LDL)/very low‐density lipoprotein (VLDL) cholesterol fraction were quantified using the AF HDL and LDL/VLDL Assay Kit (Sigma‐Aldrich, Merck Life Sciences, South Africa). Assays were performed in duplicate according to the manufacturer's protocol. Absorbance was measured at 570 nm.

The total cholesterol‐to‐HDL ratio was calculated using Equation (1):

(1) $$\text{Cholesterol}:\mathrm{HDL}=\frac{\text{total cholesterol}}{\mathrm{HDL}\;}$$

Serum triglyceride levels were measured using the Mission 3‐in‐1 Cholesterol Meter using Mission Cholesterol Test 3–1 Lipid Panel Strips (range: 65–650 mg/dL), applied to terminal serum samples.

### 2.6 Serum calcium measurements

Serum calcium levels were determined using the Calcium Colorimetric Assay Kit (Sigma‐Aldrich). Assays were performed in duplicate according to the manufacturer's protocol. Absorbance was measured at 570 nm.

### 2.7 Serum markers of inflammation

IL‐6 levels were measured using the Rat IL‐6 ELISA Kit (Invitrogen, Thermo Fisher Scientific, South Africa) following the manufacturer's instructions with serum dilution (1:2). C‐reactive protein (CRP) and SAA levels were determined using Rat CRP and Rat SAA ELISA kits (Elabscience, Biocom Africa, South Africa), respectively, according to the manufacturer's instructions. Serum samples were diluted 1:500 000 for the CRP analysis and 1:200 000 for the SAA analysis. All absorbance readings were obtained at 450 nm. IL‐6, CRP and SAA concentrations in serum samples were determined from standard curves for each enzyme‐linked immunosorbent assay (ELISA). All samples were run in duplicate.

### 2.8 Serum markers of endothelial dysfunction

Endothelial nitric oxide synthase (eNOS) and intracellular adhesion molecule‐1 (ICAM‐1) serum levels were determined using the Rat NOS3/eNOS ELISA (Elabscience Kit, Biocom Africa) and the Rat ICAM‐1/CD54 ELISA (Elabscience Kit, Biocom Africa), respectively, according to the manufacturer's instructions. Serum samples were diluted 1:10 for eNOS and 1:2 for ICAM‐1 analyses. Absorbance for all ELISA assays was measured at 450 nm. eNOS and ICAM‐1 concentrations in serum samples were determined from standard curves for each ELISA. All samples were run in duplicate.

### 2.9 Lactate dehydrogenase activity

The Lactate Dehydrogenase (LDH) Activity Assay Kit (Sigma‐Aldrich) was used to quantify LDH activity in serum samples. Serum samples were diluted 1:1000. Assays were performed in duplicate according to the manufacturer's protocol. Absorbance was measured at 450 nm.

### 2.10 Histology

After the animals were euthanized, hearts (including a section of the aorta) and livers were harvested and fixed in 4% buffered formaldehyde. Tissues were then embedded in paraffin, sectioned transversely (5 μm), and stained using hematoxylin and eosin (H&E) and von Kossa protocols.^22^ Histological sections were examined using a Leica light microscope (Leica Microsystems, Switzerland) equipped with a Leica ICC50 W digital camera. Images were captured and processed using the Leica Application Suite EZ (LAS EZ) software, version 3.4.0 (Leica Microsystems). Images were obtained at magnifications of 40×, 100×, and 200×, with consistent exposure and contrast settings applied across groups to allow direct comparison. Aortic wall thickness was quantified from H&E microimages using ImageJ (NIH). Aortic tissue was sampled from the ascending aorta, with sections cut just below the arch. Aortic wall thickness was quantified in four control and five model animals using one transverse section per animal. Using calibrated digital image analysis, total aortic wall thickness was measured at four equidistant radial positions around the vessel circumference (quadrants) and averaged to obtain one mean wall thickness value per animal. Blinded assessment was performed as described previously. Gross morphology of the livers was documented using images.

### 2.11 Statistical analysis

Data were analyzed using GraphPad Prism, version 10.2.2 (GraphPad Software, La Jolla, CA, USA). Sample size was determined using the resource equation approach, ensuring that the degrees of freedom for error remained between 10 and 20.^23^ Normality was not assumed due to small group sizes; therefore, nonparametric tests were applied. Between‐group comparisons at the study endpoint were performed using the Mann–Whitney *U* test. Within‐group paired comparisons (baseline vs. week 10) were performed using the Wilcoxon signed‐rank test. For serial whole‐blood cholesterol monitoring, values outside the device's analytical range (100–500 mg/dL, recorded as “<100” or “>500”) were treated as nonquantifiable and excluded from the calculation of mean ± standard deviation. To incorporate all animals into the longitudinal interpretation, categorical threshold analyses were additionally performed using a two‐tailed Fisher's exact test to compare the proportion of animals with cholesterol ≥100 mg/dL (including above range >500 mg/dL) versus <100 mg/dL at each time point. Statistical significance was set at *p* < 0.05.

## 3 RESULTS

### 3.1 Food intake per group and changes in weight and whole‐blood cholesterol and triglyceride levels during the feeding period

**FIGURE 2:**
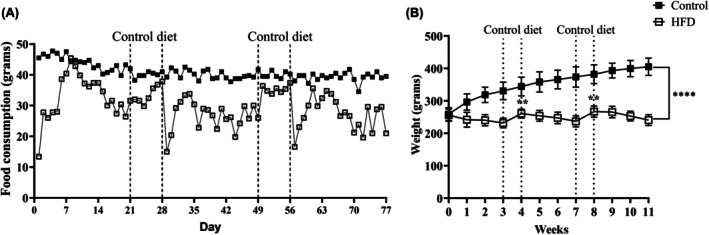
(A) Average daily food intake of the control (*n* = 8) and high‐fat‐diet (HFD) (*n* = 10) groups over 11 weeks. The control diet was introduced in the ratio of 60 to 40 (control diet to HFD diet) after week 3 (21 days) and week 7 (49 days). (B) Average weekly weights of the control (*n* = 8) and HFD (*n* = 10) groups at the beginning of each week. Vertical dashed lines indicate weeks when the HFD group received the 60:40 (control–HFD) mix. **p < 0.01 and ****p < 0.0001.

Rats were transitioned to the HFD over 3 days. On the first day of 100% HFD, intake was <20 g per cage, increased after week 1, but remained below that of the control group throughout. Notably, the control group also did not consume all of the feed provided. In the HFD group, after each 60:40 control–HFD week, reintroducing 100% HFD again decreased intake to <20 g per cage, which then partially recovered but stayed lower than that of controls (Figure 2A). Controls gained weight steadily (Figure 2B). HFD rats lost weight after initiation of the HFD, with rebounds during the control‐diet weeks (weeks 4 and 8, *p* = 0.002). The final average weight was 405.00 ± 26.61 g (control) versus 241.00 ± 16.83 g (HFD), *p* < 0.0001.

**FIGURE 3:**
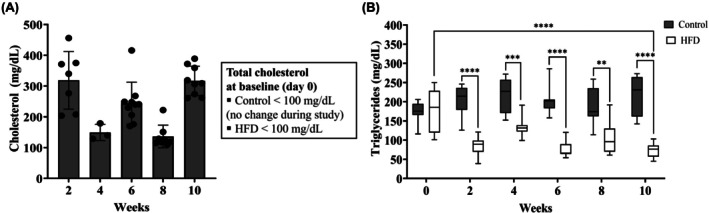
(A) Biweekly whole‐blood total cholesterol in the HFD (high‐fat diet) group measured using the Mission 3‐in‐1 Cholesterol Meter (quantifiable range: 100–500 mg/dL). Bars represent mean ± SD (standard deviation), and dots indicate individual rats with in‐range readings; control animals remained <100 mg/dL throughout (recorded as “<100 mg/dL”; see Table 1) and are therefore not plotted. (B) Biweekly whole‐blood triglyceride levels in the control (*n* = 8) and HFD (*n* = 10) groups over 10 weeks. **p < 0.01, ***p < 0.001 and ****p < 0.0001. [Correction added on 08 May 2026, after online publication: The cholesterol value at week‐10 in Figure 3A has been corrected.]

Figure 3A shows only in‐range cholesterol values for the HFD group. The frequency of out‐of‐range values and the number of animals affected at each time point are provided in Table 1. At baseline, whole‐blood cholesterol levels in both the control and HFD groups were <100 mg/dL. Cholesterol levels in the control group remained <100 mg/dL for the remainder of the study (Table 1). In the HFD group, average whole‐blood cholesterol levels increased to 318.60 ± 93.45 mg/dL by week 2 (Figure 3A). Fluctuations in the HFD group's cholesterol levels were observed during the remainder of the study, corresponding to the introduction of the control diet. A decrease in whole‐blood cholesterol levels was observed during the weeks when the control diet was introduced. At week 10, the average cholesterol level in the HFD group was 316.8 ± 47.66 mg/dL.

**TABLE 1:** Out‐of‐threshold whole‐blood cholesterol levels taken at biweekly intervals.

Time point	Group	
	Control (*n* = 8)	HFD (*n* = 10)
Baseline (day)	<100 mg/dL (8/8)	<100 mg/dL (10/10)
Week 2	<100 mg/dL (8/8)	**>500 mg/dL (3/10)**
Week 4	<100 mg/dL (8/8)	<100 mg/dL (7/10)
Week 6	<100 mg/dL (8/8)	All values within range^a^
Week 8	<100 mg/dL (8/8)	<100 mg/dL (2/10)
Week 10	<100 mg/dL (8/8)	**>500 mg/dL (1/10)**

*Note*: Blue shading indicates the measured values after the control feed was administered with the HFD in the HFD group. Out‐of‐range values above the upper threshold (>500 mg/dL) are highlighted in bold.

Abbreviation: HFD, high‐fat diet.

^a^ Mission 3‐in‐1 Cholesterol Meter Threshold: 100–500 mg/dL.

Using a categorical threshold analysis (<100 vs. ≥100 mg/dL) to incorporate out‐of‐range readings, the HFD group exhibited a significantly higher proportion of animals with cholesterol ≥100 mg/dL at weeks 2, 6, 8, and 10 compared with controls (Table 2), whereas week 4 exhibited a transient increase in below‐range readings consistent with the scheduled 60:40 control–HFD week.

**TABLE 2:** Threshold analysis of serial whole‐blood cholesterol readings (≥100 vs. <100 mg/dL) comparing control and HFD groups at each time point.

Time point	Control: ≥100 mg/dL, *n* (%)	HFD: ≥100 mg/dL, *n* (%)	Fisher's exact *p*‐value
Baseline (day 0)	0 (0%)	0 (0%)	–
Week 2	0 (0%)	10 (100%)	**<0.0001**
Week 4	0 (0%)	3 (30%)	0.2157
Week 6	0 (0%)	10 (100%)	**<0.0001**
Week 8	0 (0%)	8 (80%)	**0.00105**
Week 10	0 (0%)	10 (100%)	**<0.0001**

*Note*: Values ≥100 mg/dL include both in‐range (100–500 mg/dL) and above‐range (>500 mg/dL) meter readings; values <100 mg/dL were classified as below threshold. Percentages are based on group sizes (control, *n* = 8; HFD, *n* = 10). Values in bold font indicate significance (*p* < 0.05).

Abbreviation: HFD, high‐fat diet.

There was no significant difference in triglyceride levels between the control and HFD groups at baseline (control 172.90 ± 27.30 mg/dL vs. HFD 177.60 ± 54.70 mg/dL, *p* = 0.6798) (Figure 3B). However, a significant decrease in triglyceride levels was observed in the HFD group starting at week 2 (*p* < 0.05), and the triglyceride levels at the end of week 10 were significantly lower than baseline levels (73.90 ± 18.82 mg/dL, *p* < 0.0001).

In the HFD group, three rats had whole‐blood cholesterol levels exceeding the upper threshold of 500 mg/dL by week 2 (Table 1). Seven rats had cholesterol levels <100 mg/dL after the control diet was introduced in week 4. By week 8, two rats had cholesterol levels <100 mg/dL after the introduction of the control diet, and one rat had a cholesterol level >500 mg/dL at the end of the study (Table 1).

### 3.2 Serum cholesterol, triglyceride, and calcium levels at the end of the study

**FIGURE 4:**
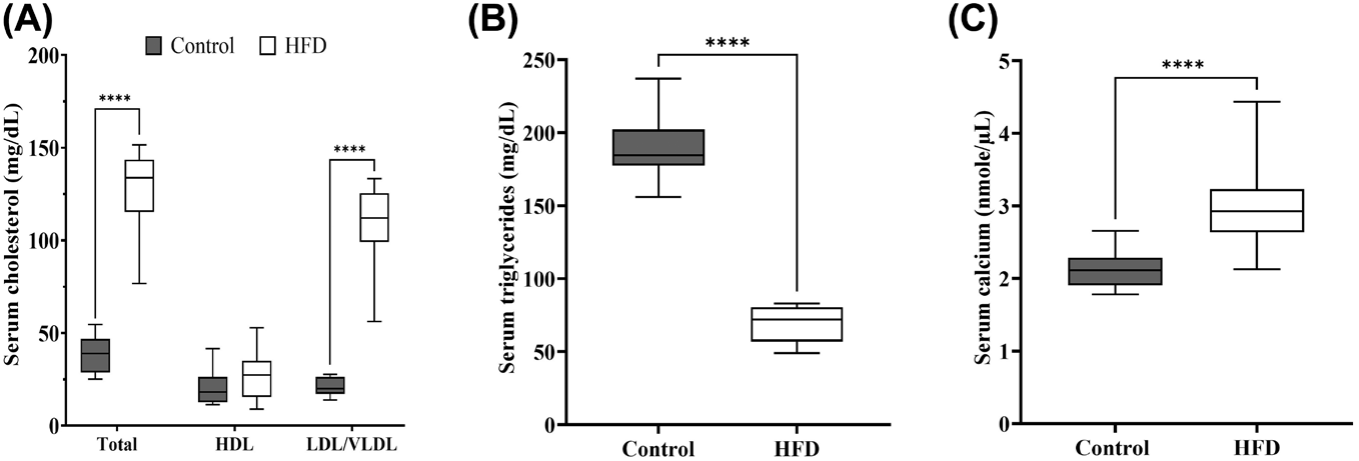
Serum levels of (A) cholesterol (total, high‐density lipoprotein [HDL], low‐density lipoprotein [LDL], and very low‐density lipoprotein [VLDL]); (B) triglycerides; and (C) calcium for the control group (*n* = 8) and high‐fat‐diet (HFD) group (*n* = 10) at the end of the 11‐week feeding period; ****p < 0.0001.

At week 11, HFD rats had higher total cholesterol than controls (HFD 127.80 ± 21.68 mg/dL vs. control 38.85 ± 10.07 mg/dL, *p* < 0.0001), with no HDL difference (HFD 27.42 ± 13.01 mg/dL vs. control 20.83 ± 10.08 mg/dL, *p* = 0.2370), yielding a higher total cholesterol‐to‐HDL ratio (HFD 5.82 ± 3.59 vs. control 2.05 ± 0.19, *p* < 0.0001) (Figure 4A). The LDL and VLDL levels were higher in the HFD group (HFD 108.40 ± 23.49 mg/dL vs. control 20.80 ± 5.01 mg/dL, *p* < 0.0001), whereas triglycerides were significantly lower in the HFD group (HFD 69.60 ± 12.36 mg/dL vs. control 190.40 ± 23.80 mg/dL, *p* < 0.0001) (Figure 4A,B). Serum calcium was higher in the HFD group (HFD 2.98 ± 0.48 nmol/μL vs. control 2.135 ± 0.26 nmol/μL, *p* < 0.0001) (Figure 4C).

### 3.3 Serum markers of inflammation, endothelial dysfunction, and LDH activity at the end of the study

**FIGURE 5:**
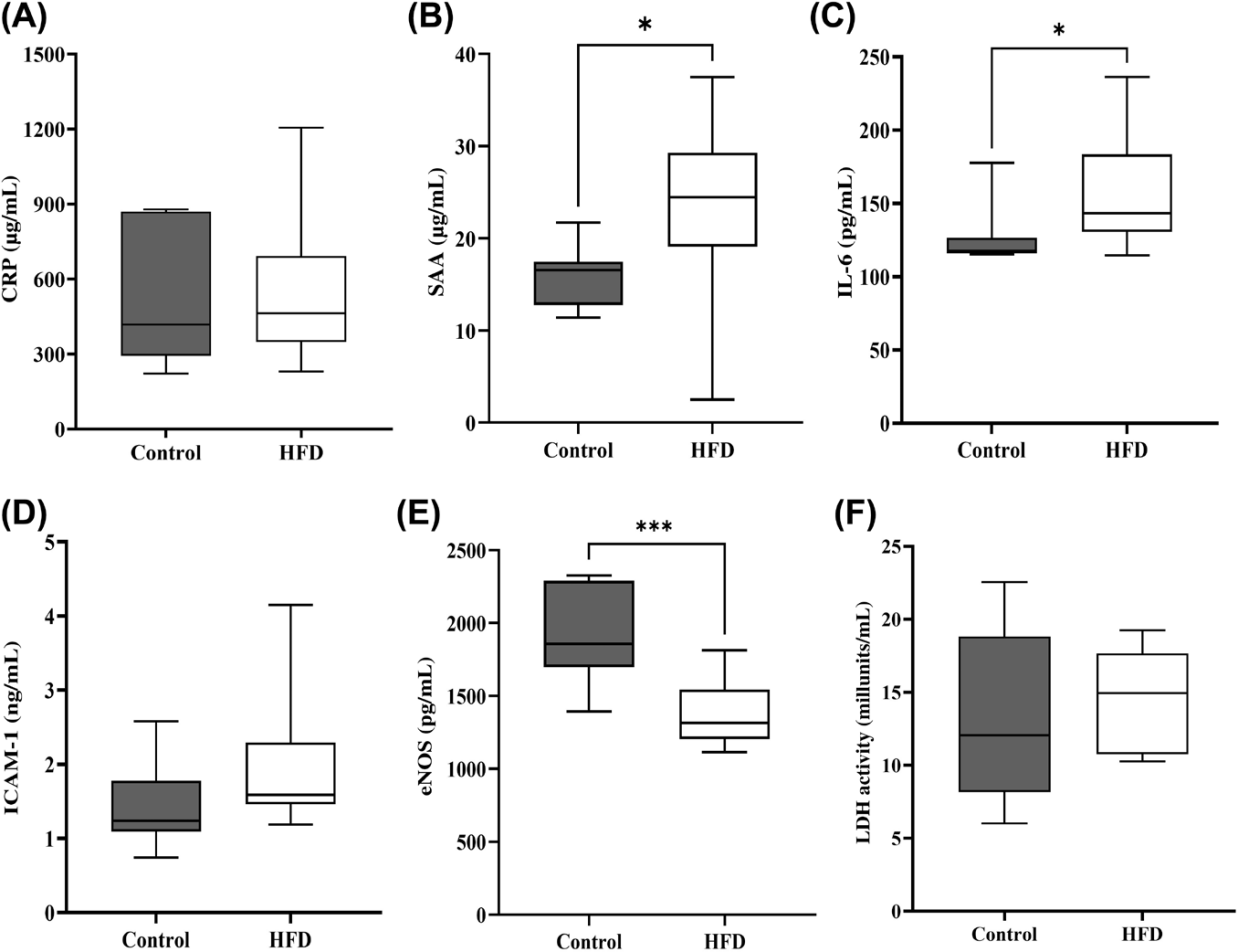
Serum levels of (A) C‐reactive protein (CRP), (B) serum amyloid A (SAA), (C) interleukin‐6 (IL‐6), (D) intracellular adhesion molecule‐1 (ICAM‐1), (E) endothelial nitric oxide synthase (eNOS), and (F) lactate dehydrogenase (LDH) activities for the control group (*n* = 8) and high‐fat‐diet (HFD) group (*n* = 10) at the end of the 11‐week feeding period; ***p < 0.001 and *p < 0.05.

CRP did not differ significantly (HFD 551.20 ± 292.90 vs. control 520.10 ± 282.90 μg/mL, *p* = 0.8412; Figure 5A). SAA was significantly higher in the HFD group (HFD 22.95 ± 9.85 vs. control 16.05 ± 3.28 μg/mL, *p* = 0.0343; Figure 5B), and IL‐6 was significantly higher in the HFD group (HFD 156.70 ± 41.30 vs. control 126.10 ± 21.26 pg/mL, *p* = 0.0464; Figure 5C). ICAM‐1 did not differ significantly between the two groups (HFD 1.977 ± 0.926 vs. control 1.448 ± 0.5764 ng/mL, *p* = 0.1273; Figure 5D). eNOS was significantly lower in the HFD group (HFD 1384 ± 222.40 vs. control 1973.00 ± 338.00 pg/mL, *p* = 0.0009; Figure 5E). Serum LDH activity did not differ between groups (HFD 14.69 ± 3.35 vs. control 12.96 ± 5.89 mU/mL, *p* = 0.3599; Figure 5F).

### 3.4 Histological changes in aorta and coronary arteries

**FIGURE 6:**
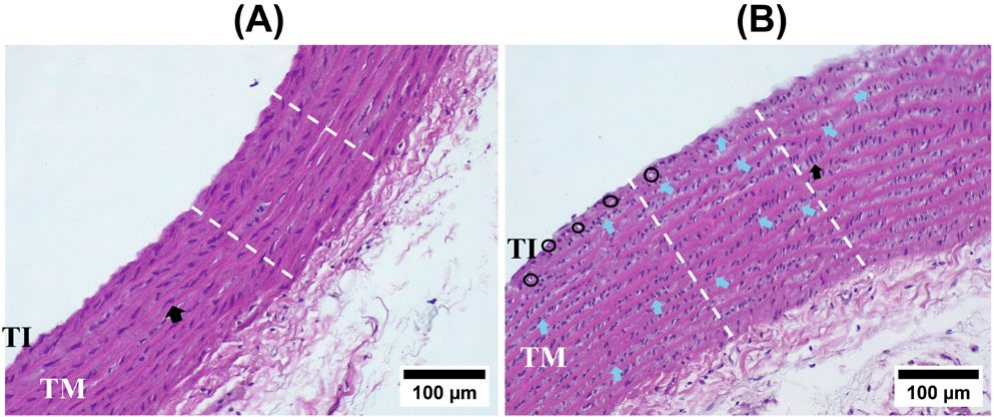
Histological comparison of the aortic wall between the control and high‐fat‐diet (HFD) groups (H&E [hematoxylin and eosin], 200×). (A) Control group showing intact tunica intima (TI) and tunica media (TM), with medial smooth‐muscle cells (black arrow) oriented parallel to the aortic lumen; no inflammatory infiltrates are present. (B) HFD aorta showing a thicker TM (vs. control), multifocal mononuclear cell infiltrates (blue arrows), and disordered smooth‐muscle orientation in the TM (black arrow). Occasional vacuolated cells are visible in the TI (circled), suggesting early foam cell‐like changes. Scale bar = 100 μm.

**FIGURE 7:**
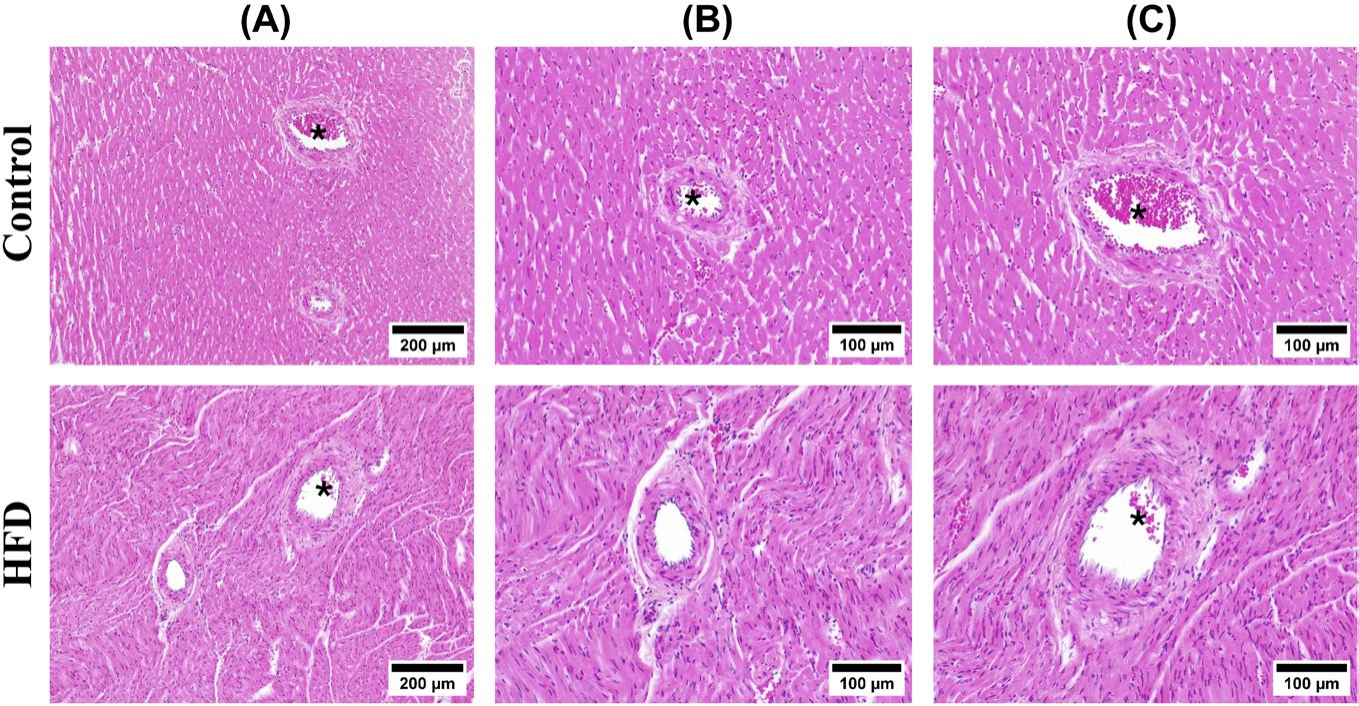
Histological comparison of coronary arteries between the control and high‐fat‐diet (HFD) groups (H&E [hematoxylin and eosin], panel A at 100×; panels B and C: Sections of the same image as in panel A at 200×). Top row: Control group showing smooth endothelial lining without inflammatory infiltrates. Bottom row: HFD group exhibiting irregular endothelial lining with focal intravascular and perivascular inflammatory cell infiltration. Scale bar = 200 μm (A) and 100 μm (B and C). *Intraluminal erythrocyte aggregates; aggregates are nonadherent to the endothelium and not occlusive.

**FIGURE 8:**
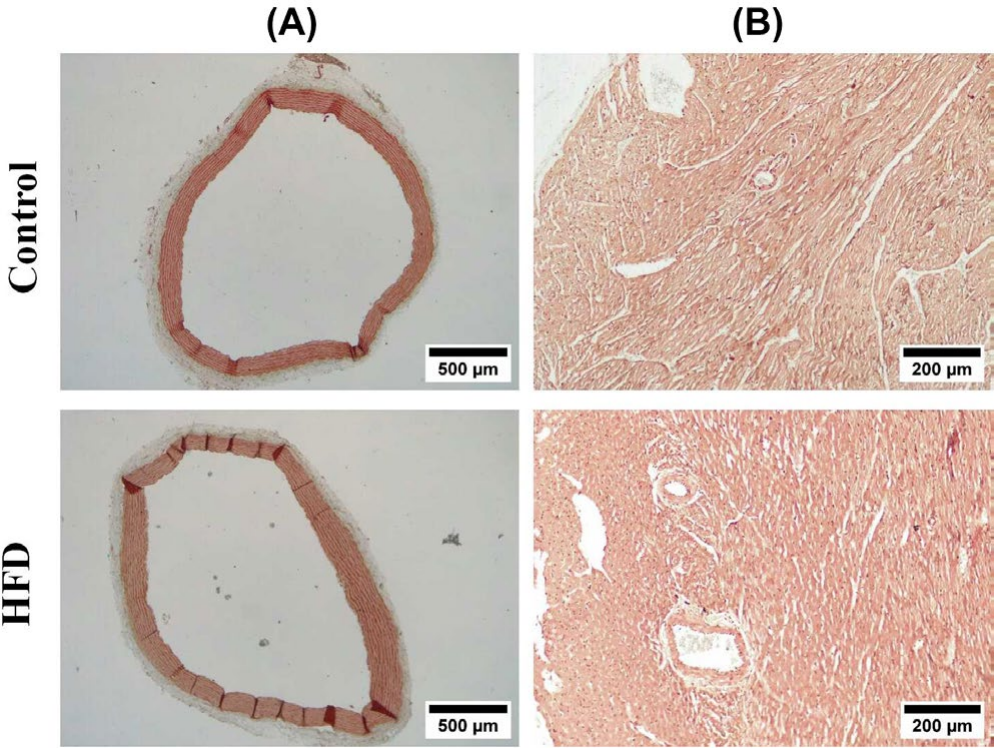
von Kossa staining of the aorta and coronary arteries in the control and high‐fat‐diet (HFD) groups (panel A: at 40×; panel B: at 100×). (A) Representative cross sections of the aorta showing the absence of vascular calcification in both control and HFD groups. (B) Representative sections of coronary arteries showing the absence of calcification in both the control and HFD groups. Scale bar = 500 μm (A) and 200 μm (B).

The aorta in controls (Figure 6A) exhibited an intact intima and media with circumferentially aligned smooth‐muscle cells (SMC) and no inflammatory infiltrates. In HFD rats (Figure 6B), the aortic wall exhibited thickening, SMC disarray, and multifocal mononuclear infiltrates. Occasional subintimal vacuolated cells with round nuclei were present, consistent with an early foam cell–like change. Aortic wall thickness was higher in the HFD group than in the controls (HFD 178.47 ± 14.76, *n* = 5, vs. control 121.17 ± 10.83, *n* = 4; *p* = 0.0159), with a large effect size (Hedges' *g* = 3.85). The coronary arteries in the controls (Figure 7A–C, top row) had a continuous, smooth endothelium and preserved wall architecture, whereas arteries from HFD‐fed animals (Figure 7A–C, bottom row) exhibited an irregular endothelial lining, focal intraluminal mononuclear infiltrates, and perivascular inflammatory cell accumulation. von Kossa staining of the aorta and coronaries (Figure 8A,B) was negative for calcification in both groups.

### 3.5 Histological changes in the liver

**FIGURE 9:**
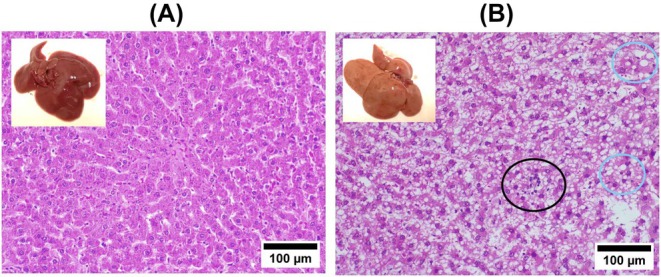
Histological and gross comparison of liver tissue between the control and high‐fat‐diet (HFD) groups (H&E [hematoxylin and eosin], 200×). (A) Control group showing preserved hepatic architecture without steatosis or inflammatory infiltrates; gross liver morphology appears normal (inset). (B) HFD group demonstrating steatosis (blue circles) and lobular inflammatory cell infiltration (black circle), with altered gross liver morphology shown in the inset. Scale bar = 100 μm.

Controls (Figure 9A) had normal gross and histological appearances. HFD rats (Figure 9B) exhibited micro‐ and macrovesicular steatosis, accompanied by lobular inflammatory infiltrates; gross inspection was consistent with the presence of steatosis.

## 4 DISCUSSION

Nongenetic rat models combining a HFD with vitamin D (with or without PTU) are widely used to induce atherogenesis.^12^, ^13^, ^15^, ^18^, ^19^, ^20^, ^24^ To balance disease induction with welfare, we used a moderate‐dose regimen (vitamin D_3_ 200 000 IU/kg diet plus PTU 0.2%) and incorporated intermittent mixed‐diet weeks. This strategy avoided mortality and helped stabilize body condition, but the HFD group exhibited lower and more variable food intake than controls, with body weight tracking intake and improving during the mixed‐diet weeks, supporting intake‐linked weight dynamics rather than clear evidence of severe toxicity. Particularly, the digestible energy density of the HFD (14.60 MJ/kg) was only marginally higher than the control diet (14.20 MJ/kg), indicating that the lower final body weight in the HFD group is more consistent with reduced/variable intake and diet tolerability than with a lower caloric density.

Lipid endpoints confirmed model induction. At the end of the study, serum total cholesterol and LDL/VLDL levels were significantly higher in the HFD group (*p* < 0.0001), whereas triglyceride levels were significantly lower (*p* < 0.0001). Whole‐blood cholesterol fluctuated with diet cycling (>240 mg/dL during 100% HFD weeks and <200 mg/dL with mixed‐diet reintroduction). According to the 2018 guidelines on blood cholesterol management published in the *Journal of the American College of Cardiology*, total cholesterol <200 mg/dL is considered desirable, and ≥240 mg/dL high in adults,^25^ supporting the translational relevance of the observed excursions. The elevated total cholesterol‐to‐HDL ratio (>5:1 in HFD) further supports a pro‐atherogenic profile.

Blood samples for lipid measurements were collected in the nonfasted state to minimize stress and avoid prolonged food withdrawal; therefore, triglyceride concentrations should be interpreted as nonfasted values. Serum triglycerides were significantly lower in the HFD group during and at the end of the study (*p* < 0.0001), with comparable baseline levels between groups. Because triglycerides are sensitive to prandial state, nonfasted sampling is expected to yield greater variability and can produce higher baseline values than fasting measurements; importantly, both groups were sampled under the same conditions, supporting the validity of between‐group comparisons over time. Lower circulating triglycerides in the setting of marked hypercholesterolemia have been reported in cholesterol‐ and bile‐salt‐based rat diets and may reflect broader shifts in lipid handling and distribution. To support this, Wang et al.^26^ showed that cholesterol/bile salt feeding increased hepatic cholesterol and triglyceride accumulation while lowering serum triglycerides, consistent with altered hepatic lipid trafficking during early dyslipidemia. In the present study, hepatic steatosis on histology together with significantly elevated VLDL/LDL cholesterol and reduced triglycerides aligns with a cholesterol‐dominant dyslipidemia pattern rather than the hypertriglyceridemia phenotype often seen in obesity‐driven models. Minor fluctuations in serial lipid values may reflect weeks during which the HFD group received a 60:40 control–HFD mixture. Because these dilution weeks temporarily reduce the atherogenic dietary load, they may contribute to short‐term attenuation/variability in serial lipid measurements. Therefore, lipid data are interpreted as overall trajectories across the study, and key endpoints are confirmed using terminal serum lipid analysis when the animals are euthanized.

Increased hepatic triglyceride levels contribute to the development of hepatic steatosis.^27^ Whereas hepatic triglyceride levels were not explicitly measured in the current study, liver histology was examined to evaluate whether the HFD induced hepatic steatosis as part of the systemic metabolic response. HFD‐fed rats exhibited micro‐ and macrovesicular steatosis and lobular inflammatory infiltrates, consistent with fatty liver (simple steatosis) within the spectrum of metabolic dysfunction–associated steatotic liver disease (MASLD). MASLD is an early, independent risk factor for atherosclerosis^28^ and is linked to obesity and type 2 diabetes.^29^, ^30^ In rodents, a HFD induces hepatic steatosis even without weight gain.^31^ Compared to dietary fructose, dietary fat and cholesterol are more potent drivers of MASLD development and progression in Sprague–Dawley rats.^32^ Similar observations have been observed in human populations, where an increasing proportion of nonoverweight individuals are affected by MASLD, highlighting dyslipidemia as an important independent risk factor.^33^

Rats in the HFD group had significantly higher serum calcium levels (*p* < 0.0001) compared to the control group. However, no calcification was found in the aorta or coronary arteries on histology. The higher serum calcium levels may reflect the vitamin D supplementation included in the HFD, as vitamin D increases intestinal calcium absorption.^34^

Three inflammatory markers, CRP, IL‐6, and SAA were measured in this study. No significant differences in CRP levels were observed between control and HFD‐fed rats, although both groups exhibited high serum CRP concentrations (averaging >500 μg/mL). In healthy, pathogen‐free rats, baseline CRP typically ranges from 300 to 600 μg/mL.^35^ These values are significantly higher than those observed in humans, where baseline CRP levels are usually <3 mg/L (equivalent to <3 μg/mL).^36^ This species‐specific difference explains why CRP values that appear elevated relative to human physiology are within the expected physiological range for rats. A previous study in which Wistar rats were fed a HFD for the induction of atherosclerosis also found no increase in CRP.^37^ The HFD led to significant increases (*p* < 0.05) in serum IL‐6 and SAA levels compared to the control group. IL‐6 is a pro‐inflammatory cytokine mainly expressed in leukocytes, endothelial cells, and vascular SMCs.^6^, ^38^ This systemic inflammation mirrors the early stages of atherogenesis in humans, where IL‐6 and acute‐phase proteins increase, predicting cardiovascular risk. IL‐6 signaling plays a key role in the pathogenesis of atherosclerosis, with increased IL‐6 levels resulting from oxidative stress and vascular injury.^7^, ^39^ IL‐6 reliably predicts cardiovascular events and increases in early atherosclerosis stages, independent of CRP.^40^ Consistent with human data linking persistently elevated SAA to chronic inflammation and cardiovascular risk,^4^, ^41^ SAA concentrations were significantly higher in rats fed a HFD compared to the control group.

SAA can contribute to endothelial dysfunction by modulating eNOS and adhesion molecule pathways, including effects on eNOS and ICAM‐1 expression.^5^, ^42^, ^43^ In this context, the observed increases in IL‐6 and SAA together with lower circulating eNOS are consistent with early endothelial perturbation in this model. Particularly, because eNOS was measured in serum using ELISA, it should be interpreted as a supportive, indirect marker of altered nitric oxide signaling rather than a direct measure of vascular eNOS expression or NO bioavailability. In comparable rat models, interventions that attenuate atherogenesis have been reported to lower inflammatory cytokines and restore aortic eNOS/NO signaling,^24^ underscoring the relevance of this pathway. Although ICAM‐1 did not differ significantly between groups, the upward trend may be consistent with early endothelial activation. Because this model reflects an early/pre‐plaque stage, systemic soluble ICAM‐1 may not yet show a clear between‐group separation. Future studies should directly evaluate vascular ICAM‐1 expression (e.g., immunohistochemistry of aortic sections) to better characterize endothelial activation in situ.

The biochemical pattern (elevated IL‐6 and SAA with lower circulating eNOS) co‐occurred with histological evidence of early vascular remodeling and inflammation. HFD‐fed rats exhibited aortic thickening with disarrayed SMCs, multifocal mononuclear infiltrates, and occasional subintimal vacuolated cells suggestive of early foam cell–like change, alongside coronary endothelial irregularities and intra−/perivascular inflammatory cell accumulation. von Kossa staining confirmed the absence of vascular calcification, supporting pre‐calcified, pre‐plaque remodeling rather than advanced atheromatous lesions.

## 5 LIMITATIONS OF THE STUDY

Food intake was estimated per pair‐housed cage, which may overestimate the true intake due to spillage; individual housing would improve precision, but this approach conflicts with welfare considerations. The alternating HFD–control mix prevented excessive weight loss and mortality but introduced lipid variability—a “push–pull” dynamic that also reflects real‐world nonadherence. Biomarkers were measured only at the endpoint; future work should include longitudinal assessments of IL‐6, SAA, and eNOS to better capture the temporal dynamics and physiological adaptations during the intervention. Thyroid status (TSH/T4/T3) and vitamin D status [25(OH)D] were not measured. Including these endpoints would provide objective confirmation that PTU and vitamin D achieved the intended physiological perturbations, enabling better cross‐study standardization.

## 6 CONCLUSION

A HFD plus vitamin D_3_ (200 000 IU/kg diet) plus PTU (0.2%) rat model developed hypercholesterolemia (LDL‐dominant), systemic inflammation (higher IL‐6 and SAA), evidence of early endothelial perturbation (lower circulating eNOS), and aortic thickening. Coronary arteries exhibited endothelial irregularities and perivascular inflammation. Steatosis confirmed systemic metabolic engagement, whereas the absence of vascular calcification localized the pathology to pre‐plaque, pre‐calcific stages. No mortality occurred.

## AUTHOR CONTRIBUTIONS

**Angélique Lewies:** Conceptualization; formal analysis; funding acquisition; investigation; writing – original draft. **Vitaris Kodogo:** Investigation; writing – review and editing. **Johannes Frederik Wentzel:** Formal analysis; visualization; writing – original draft. **Francis Edwin Smit:** Conceptualization; writing – review and editing.

## FUNDING INFORMATION

This study was funded by a National Research Foundation Thuthuka Grant (grant number 129530).

## CONFLICT OF INTEREST STATEMENT

The authors declare that this study was conducted in the absence of any commercial or financial relationships that could be construed as a potential conflict of interest.

## ETHICS STATEMENT

All procedures were approved by the interfaculty Animal Research Ethics Committee (UFS‐AED/2021/0003) and the Environment and Biosafety Research Ethics Committee (UFS‐ESD2021/0217) at the UFS.

## Supporting information


**Table S1.** Composition of Specialty Feeds meat‐free rat and mouse diet.
**Table S2**. Specialty Feeds cholesterol‐enriched meat‐free rat and mouse diet (diet SF21‐049).

## Data Availability

Data are available upon reasonable request from the corresponding author.
