# Supplementary material for: A high‐fat diet with vitamin D and propylthiouracil produces a pro‐atherogenic phenotype in rats
Source: Animal Model Exp Med. 2026 Apr 1;9(5):1026–36. doi: 10.1002/ame2.70178 (PMC13331555; doi:10.1002/ame2.70178)
Supplement: Supplementary file 1 — Table S1. Composition of Specialty Feeds meat‐free rat and mouse diet. Table S2. Specialty Feeds cholesterol‐enriched meat‐free rat and mouse diet (diet SF21‐049). [file AME2-9-1026-s001.docx]

**Table S1: Composition of Specialty Feeds Meat-Free Rat & Mouse Diet**

| **Category** | **Parameter** | **Calculated Value** |
| --- | --- | --- |
| **Ingredients** | A fixed formula ration using the following ingredients:  Wheat, Barley, Lupins, Soya meal, Fish meal, Mixed vegetable oils, Canola oils, Salt, Calcium carbonate, Dicalcium phosphate, Magnesium oxide, and a Vitamin and trace mineral premix | Calculated data, presented here, uses information from the typical raw material composition |
| **Nutrition Parameters** | Protein | 19.00 % |
|  | Total fat | 4.60 % |
|  | Total carbohydrate | 59.90 % |
|  | Crude fibre | 5.20 % |
|  | Acid-detergent fibre (ADF) | 7.70 % |
|  | Neutral-detergent fibre (NDF) | 15.50 % |
|  | Digestible energy | 14.20 MJ kg⁻¹ |
|  | % Total calculated energy from protein | 23.00 % |
|  | % Total calculated energy from lipids | 12.00 % |
| **Total Minerals** | Calcium | 0.80 % |
|  | Phosphorus | 0.70 % |
|  | Magnesium | 0.20 % |
|  | Sodium | 0.20 % |
|  | Potassium | 0.80 % |
|  | Sulphur | 0.20 % |
|  | Iron | 200 mg kg⁻¹ |
|  | Copper | 24 mg kg⁻¹ |
|  | Iodine | 0.50 mg kg⁻¹ |
|  | Manganese | 114 mg kg⁻¹ |
|  | Cobalt | 0.60 mg kg⁻¹ |
|  | Zinc | 90 mg kg⁻¹ |
|  | Molybdenum | 1.20 mg kg⁻¹ |
|  | Selenium | 0.40 mg kg⁻¹ |
|  | Cadmium | 0.05 mg kg⁻¹ |
| **Total Vitamins** | Vitamin A (retinol) | 10 950 IU kg⁻¹ |
|  | Vitamin D₃ (Cholecalciferol) | 2 000 IU kg⁻¹ |
|  | Vitamin E (a Tocopherol acetate) | 110 mg kg⁻¹ |
|  | Vitamin K (Menadione) | 20 mg kg⁻¹ |
|  | Vitamin B₁ (Thiamine) | 80 mg kg⁻¹ |
|  | Vitamin B₂ (Riboflavin) | 30 mg kg⁻¹ |
|  | Niacin (Nicotinic acid) | 145 mg kg⁻^1^ |
|  | Vitamin B₆ (Pyridoxine) | 28 mg kg⁻¹ |
|  | Pantothenic acid | 60 mg kg⁻¹ |
|  | Biotin | 410 µg kg⁻¹ |
|  | Folic acid | 5 mg kg⁻¹ |
|  | Vitamin B₁₂ (Cyanocobalamin) | 150 µg kg⁻¹ |
|  | Choline | 1 640 mg kg⁻¹ |
|  | Vitamin C & Inositol | No data |
| **Amino Acids** | Valine | 0.86 % |
|  | Leucine | 1.40 % |
|  | Isoleucine | 0.80 % |
|  | Threonine | 0.70 % |
|  | Methionine | 0.30 % |
|  | Cysteine | 0.30 % |
|  | Lysine | 0.90 % |
|  | Phenylalanine | 0.90 % |
|  | Tyrosine | 0.70 % |
|  | Tryptophan | 0.20 % |
|  | Histidine | 0.50 % |
|  | Taurine | 83.4 mg kg⁻¹ |
| **Fatty Acid Composition** | Myristic Acid 14:0 | 0.03 % |
|  | Palmitic Acid 16:0 | 0.54 % |
|  | Stearic Acid 18:0 | 0.14% |
|  | Palmitoleic Acid 16:1 | 0.01% |
|  | Oleic Acid 18:1 | 1.96% |
| **Fatty Acid Composition (continued)** | Gadoleic Acid 20:1 | 0.03% |
|  | Linoleic Acid 18:2 n6 | 1.41% |
|  | α-Linolenic Acid 18:3 n3 | 0.31% |
|  | Arachidonic Acid 20:4 n6 | 0.01% |
|  | EPA 20:5 n3 | 0.02% |
|  | DHA 22:6 n3 | 0.05% |
|  | Total n3 | 0.38% |
|  | Total n6 | 1.42% |
|  | Total Monounsaturated Fats | 2.06% |
|  | Total Polyunsaturated Fats | 1.78% |
|  | Total Saturated Fats | 0.76% |

**Table S2: Specialty Feeds Cholesterol-Enriched Meat-Free Rat & Mouse Diet (Diet SF21-049)**

| **Category** | **Parameter** | **Calculated Value** |
| --- | --- | --- |
| **Ingredients** | Meat Free Rat and Mouse 943 g kg⁻¹ | Calculated data, presented here, uses information from the typical raw material composition |
|  | Cholesterol 20 g kg⁻¹ |  |
|  | Lard 30 g kg⁻¹ |  |
|  | Cholic acid 5 g kg⁻¹ |  |
|  | Propylthiouracil 2 g kg⁻¹ |  |
|  | Vitamin D_3_(500 000 IU g⁻¹) 0.4 g kg⁻¹ |  |
| **Nutrition Parameters** | Protein | 18.00 % |
|  | Total fat | 7.40 % |
|  | Total carbohydrate | 56.50 % |
|  | Crude fibre | 4.60 % |
|  | Acid-detergent fibre (ADF) | 6.90 % |
|  | Neutral-detergent fibre (NDF) | 14.20 % |
|  | Digestible energy | 14.60 MJ kg⁻¹ |
|  | % Calculated digestible energy from protein | 20.00% |
|  | % Calculated digestible energy from lipids | 18.50% |
| **Total Minerals** | Calcium | 0.75 % |
|  | Phosphorus | 0.64 % |
|  | Magnesium | 0.19 % |
|  | Sodium | 0.17 % |
|  | Potassium | 0.17 % |
|  | Sulphur | 0.16 % |
|  | Iron | 200 mg kg⁻¹ |
|  | Copper | 23 mg kg⁻¹ |
|  | Iodine | 0.50 mg kg⁻¹ |
|  | Manganese | 107 mg kg⁻¹ |
|  | Cobalt | 0.60 mg kg⁻¹ |
|  | Zinc | 90 mg kg⁻¹ |
|  | Molybdenum | 1.10 mg kg⁻¹ |
|  | Selenium | 0.30 mg kg⁻¹ |
|  | Cadmium | 0.04 mg kg⁻¹ |
| **Total Vitamins** | Vitamin A (retinol) | 10 310 IU kg⁻¹ |
|  | Vitamin D₃ (Cholecalciferol) | 201 900 IU kg⁻¹ |
|  | Vitamin E (a Tocopherol acetate) | 106 mg kg⁻¹ |
|  | Vitamin K (Menadione) | 19 mg kg⁻¹ |
|  | Vitamin B₁ (Thiamine) | 80 mg kg⁻¹ |
|  | Vitamin B₂ (Riboflavin) | 30 mg kg⁻¹ |
|  | Niacin (Nicotinic acid) | 140 mg kg⁻^1^ |
|  | Vitamin B₆ (Pyridoxine) | 26 mg kg⁻¹ |
|  | Pantothenic acid | 56 mg kg⁻¹ |
|  | Biotin | 390 µg kg⁻¹ |
|  | Folic acid | 5 mg kg⁻¹ |
|  | Vitamin B₁₂ (Cyanocobalamin) | 140 µg kg⁻¹ |
|  | Choline | 1 310 mg kg⁻¹ |
|  | Vitamin C & Inositol | No data |
| **Amino Acids** | Valine | 0.82 % |
|  | Leucine | 1.34 % |
|  | Isoleucine | 0.75 % |
|  | Threonine | 0.65 % |
|  | Methionine | 0.25 % |
|  | Cysteine | 0.31 % |
|  | Lysine | 0.89 % |
|  | Phenylalanine | 0.84 % |
|  | Tyrosine | 0.67 % |
|  | Tryptophan | 0.20 % |
|  | Histidine | 0.49 % |
| **Fatty Acid Composition** | Myristic Acid 14:0 | 0.07 % |
|  | Palmitic Acid 16:0 | 1.27% |
|  | Stearic Acid 18:0 | 0.65% |
|  | Palmitoleic Acid 16:1 | 0.06% |
|  | Oleic Acid 18:1 | 2.81% |
|  | Gadoleic Acid 20:1 | 0.05% |
| **Fatty Acid Composition (continued)** | Linoleic Acid 18:2 n6 | 1.66% |
|  | α-Linolenic Acid 18:3 n3 | 0.32% |
|  | Arachidonic Acid 20:4 n6 | Trace |
|  | EPA 20:5 n3 | 0.02% |
|  | DHA 22:6 n3 | 0.05% |
|  | Total n3 | 0.31% |
|  | Total n6 | 1.68% |
|  | Total Monounsaturated Fats | 2.94% |
|  | Total Polyunsaturated Fats | 2.09% |
|  | Total Saturated Fats | 2.07% |
